# Rapid Regulatory T-Cell Response Prevents Cytokine Storm in CD28 Superagonist Treated Mice

**DOI:** 10.1371/journal.pone.0004643

**Published:** 2009-02-27

**Authors:** Tea Gogishvili, Daniela Langenhorst, Fred Lühder, Fernando Elias, Karin Elflein, Kevin M. Dennehy, Ralf Gold, Thomas Hünig

**Affiliations:** 1 Institute for Virology and Immunobiology, University of Würzburg, Würzburg, Germany; 2 Institute for Multiple Sclerosis Research, University of Göttingen and Gemeinnützige Hertie-Stiftung, Göttingen, Germany; 3 Department of Immunology, Institute for Cell Biology, Eberhard Karls University, Tübingen, Germany; 4 Department of Neurology at St. Josef Hospital, Ruhr University Bochum, Bochum, Germany; New York University School of Medicine, United States of America

## Abstract

Superagonistic CD28-specific monoclonal antibodies (CD28SA) are highly effective activators of regulatory T-cells (Treg cells) in rats, but a first-in-man trial of the human CD28SA TGN1412 resulted in an unexpected cytokine release syndrome. Using a novel mouse anti-mouse CD28SA, we re-investigate the relationship between Treg activation and systemic cytokine release. Treg activation by CD28SA was highly efficient but depended on paracrine IL-2 from CD28SA-stimulated conventional T-cells. Systemic cytokine levels were innocuous, but depletion of Treg cells prior to CD28SA stimulation led to systemic release of proinflammatory cytokines, indicating that in rodents, Treg cells effectively suppress the inflammatory response. Since the human volunteers of the TGN1412 study were not protected by this mechanism, we also tested whether corticosteroid prophylaxis would be compatible with CD28SA induced Treg activation. We show that neither the expansion nor the functional activation of Treg cells is affected by high-dose dexamethasone sufficient to control systemic cytokine release. Our findings warn that preclinical testing of activating biologicals in rodents may miss cytokine release syndromes due to the rapid and efficacious response of the rodent Treg compartment, and suggest that polyclonal Treg activation is feasible in the presence of antiphlogistic corticosteroid prophylaxis.

## Introduction

“Natural” regulatory T-cells (Treg-cells), which leave the thymus as functional MHC class II-restricted suppressor cells, are essential for the prevention of autoimmunity and of overshooting immune responses to pathogens [1]. Manipulating the size and activity of the Treg compartment has, accordingly, become an attractive strategy in the control of immunopathology [2]–[7]. The Treg repertoire is highly diverse and is thought to be biased towards self recognition [8], thereby allowing the activation of protective Treg functions by self-antigens, including tissue-specific antigens, presented at sites of inflammation and in secondary lymphatic tissue. It is the aim of therapeutic strategies employing polyclonal Treg cell activation to dispatch clones from the activated Treg pool which recognize tissue or microbial antigens in the inflamed tissues, installing specific protection on site while allowing the remaining Treg population to return to a resting state.

The size and activity of the Treg compartment is crucially dependent on signals derived from the T-cell antigen receptor (TCR, for recognition of relevant target antigens), the high affinity IL-2R (CD25/CD122/CD132) constitutively expressed by Treg cells (for survival, fitness, and induction of suppressive activity [9]–[11]), and CD28 (required in *cis* for Treg generation and activation, and in *trans* for the production of IL-2 by conventional CD4 T-cells [12]–[16]). Accordingly, IL-2 [4], [5], and stimulatory CD28-specific mAb, so-called CD28 superagonists (CD28SA) [5], [6], [17] have been used in various rodent models for Treg-based interference with a autoimmune and inflammatory model diseases. In particular, we and others have shown that the rat CD28-specific superagonistic mAb JJ316 is highly effective *in vivo* in expanding the size and enhancing the activity of the Treg compartment [17]–[19], leading to substantial therapeutic success in rat models of autoimmunity and inflammation (reviewed in [6]).

In contrast to the benign and anti-inflammatory behaviour of the rat-specific CD28SA JJ316, the fully humanized human-CD28-specific superagonistic mAb TGN1412 induced a life-threatening cytokine release syndrome during a first-in-man trial [20], despite being well tolerated in human primates expressing CD28 molecules which bind TGN1412 with the same affinity as their human counterparts [21].

The TGN1412 trial not only raises questions about the predictive value of toxicity studies conducted in rodents and even in closely related primate species, but, more specifically, also about the relationship between the induction of toxic cytokine release by CD28SA on one side, and their ability to mediate the desired effect of polyclonal Treg activation on the other.

We have recently developed a mouse anti-mouse CD28-specific superagonistic mAb, called D665, which fully reproduces the epitope-function relationship previously described for superagonistic antibodies specific for rat and human CD28 [22]. Here, we make use of the genetic tools provided by the mouse system to investigate the mechanism by which CD28SA expand Treg cells in the rodent immune system without causing systemic cytokine release, and to ask whether pharmacological suppression of cytokine release would interfere with CD28SA-mediated Treg activation.

## Results

### CD28SA D665 expands and activates Treg cells *in vivo*

**Figure 1:**
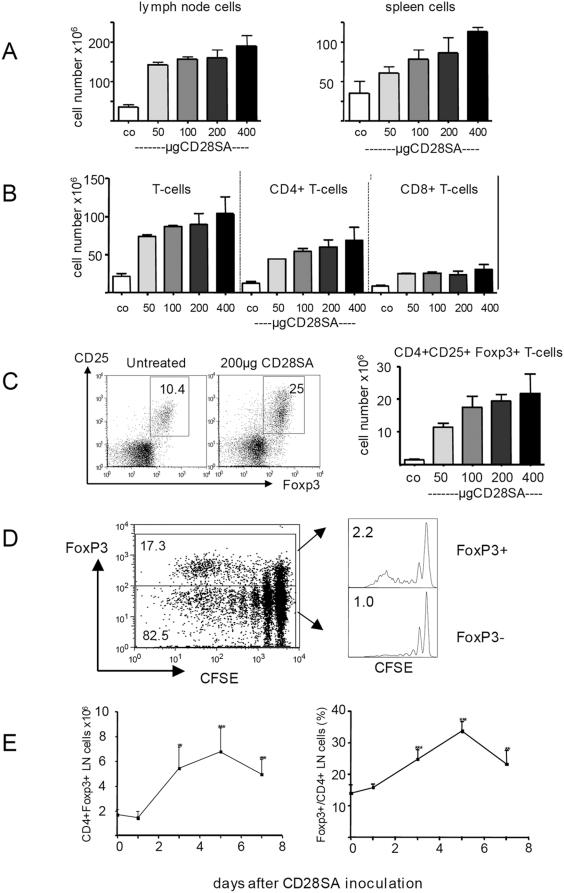
T-cell responses to mouse CD28SA D665 *in vivo*. (A) Cell numbers in pooled lymph nodes and spleen, d3. (B) T-cell subsets in lymph nodes. (C) Frequency (%) of CD25+Foxp3+ Treg cells among CD4 lymph node T-cells (left) and their absolute numbers in pooled lymph nodes (right). (D) Cell division of conventional and regulatory CD4 T-cells, d3 after stimulation with 100 µg CD28SA. 10^7^ purified CFSE-labeled CD4 T-cells were transferred i.v. on day −1. Average numbers of cell divisions for Foxp3-positive and –negative subsets (histogram inserts), and percentages of recovered subsets (dot plot inserts) are indicated. (E) Kinetics of absolute Treg cell numbers in lymph nodes (left), and of percentage among CD4 cells (right). *** indicates p<0.0001 as compared to untreated mice (d0).

C57BL/6 mice were injected with increasing doses of mAb D665, a mouse anti-mouse CD28-specific mAb of the IgG1 subclass with superagonistic properties [22]. Spleen and lymph node cells were analyzed 3 days later by flow cytometry. As shown in Fig. 1A, total cellularity increased in a dose-dependent fashion up to four-fold of control values. Most of the T-cell expansion occurred in the CD4 T-cell compartment (Fig. 1B). Intracellular staining for Foxp3 revealed that the most dramatic increase in cell number was observed in the Treg subset (Fig. 1C). As shown by monitoring of CFSE-labeled CD4 T-cells (Fig. 1D), this disproportionate increase of Treg cells is, at least in part, due to enhanced proliferation of Foxp3^+^ cells. A kinetic analysis (Fig. 1E) revealed that expansion of Treg cells peaked on day 3–5 after application of the CD28SA. Thus at the height of the response, the CD4 T-cell compartment of CD28SA stimulated mice contained up to 30% Treg cells, and up to tenfold more Treg cells than found in control mice. Transfer experiments using Treg-depleted CD4 T-cells revealed that this dramatic increase in Treg number is the result of expansion of pre-existing Treg cells, rather than conversion from “conventional” CD4 T-cells [17] (and unpublished data).

**Figure 2:**
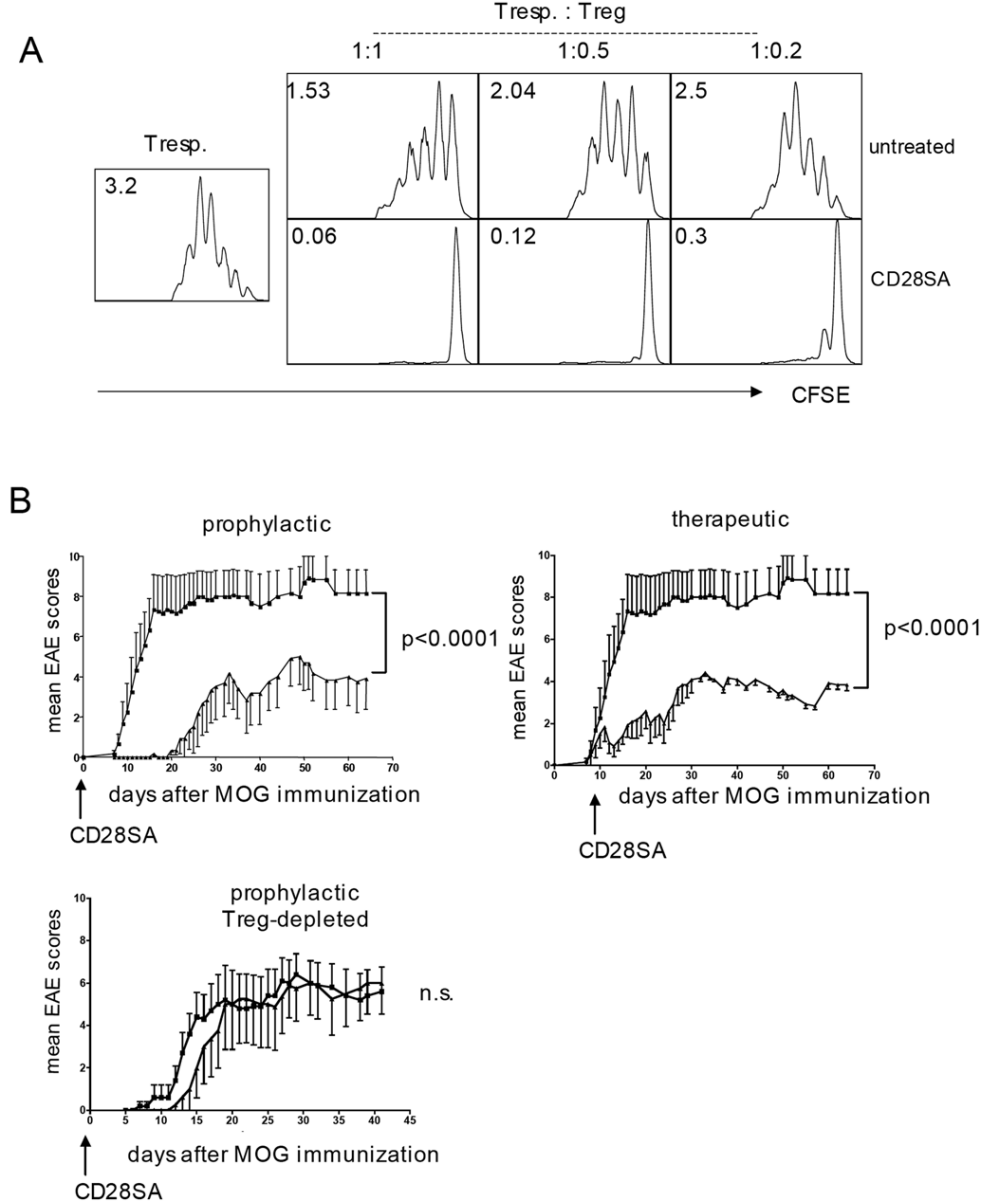
Suppressive activity of CD28SA stimulated Treg cells. (A) *In vivo* stimulation with CD28SA increases potency of Treg cells *in vitro*. CFSE-stimulated indicator CD4 T-cells were cocultured with purified Treg cells from naïve or d -3 CD28SA stimulated mice at the ratios indicated, and stimulated as given in Methods. Cell division was measured on d3, and average numbers of divisions are given as inserts. (B) CD28SA stimulation suppresses EAE by activating the Treg compartment. Left: prophylactic, middle: therapeutic treatment. Right: Treg depletion abolishes therapeutic effect. 50 µg of CD28SA were applied and EAE was read as given in Methods.

We also tested the suppresive activity of the expanded CD4^+^CD25^+^ cells recovered from CD28SA-stimulated mice *in vitro* using purified CFSE-labeled CD4^+^CD25^−^ cells as responders, and irradiated APC and anti-CD3 as a proliferative stimulus. As shown in Fig. 2A, CD4^+^CD25^+^ cells from CD28SA stimulated mice had a more than fivefold higher suppressive activity on a per cell basis than those from control mice, adding functional activation to numeric increase in the Treg-promoting effect of CD28SA.

### CD28SA interferes with EAE by activating Treg cells

To test for the therapeutic efficacy of CD28SA-mediated Treg activation, experimental autoimmune encephalomyelitis (EAE), a mouse model for multiple sclerosis, was induced by immunization with a myelin oligodendrocyte glycoprotein (MOG) derived peptide. Fig. 2B shows that CD28SA application ameliorated disease development in both, a prophylactic and a therapeutic setting. We also asked whether as hypothesized, the beneficial effect of CD28SA therapy was Treg cell dependent by first depleting CD25 expressing (i.e. Treg-) cells with the rat anti-mouse CD25 specific mAb PC61. Fig. 2B shows that thorough depletion of Treg cells (less than 1% of CD4 cells in peripheral blood, not shown), ablated the therapeutic activity of D665. This result strongly supports our notion that the CD28SA suppressed EAE by transient polyclonal activation of Treg cells, in agreement with previous work published in rat EAE [19], [23].

### CD28SA driven CD4 T(reg)-cell proliferation depends on IL-2 produced by bystander cells

**Figure 3:**
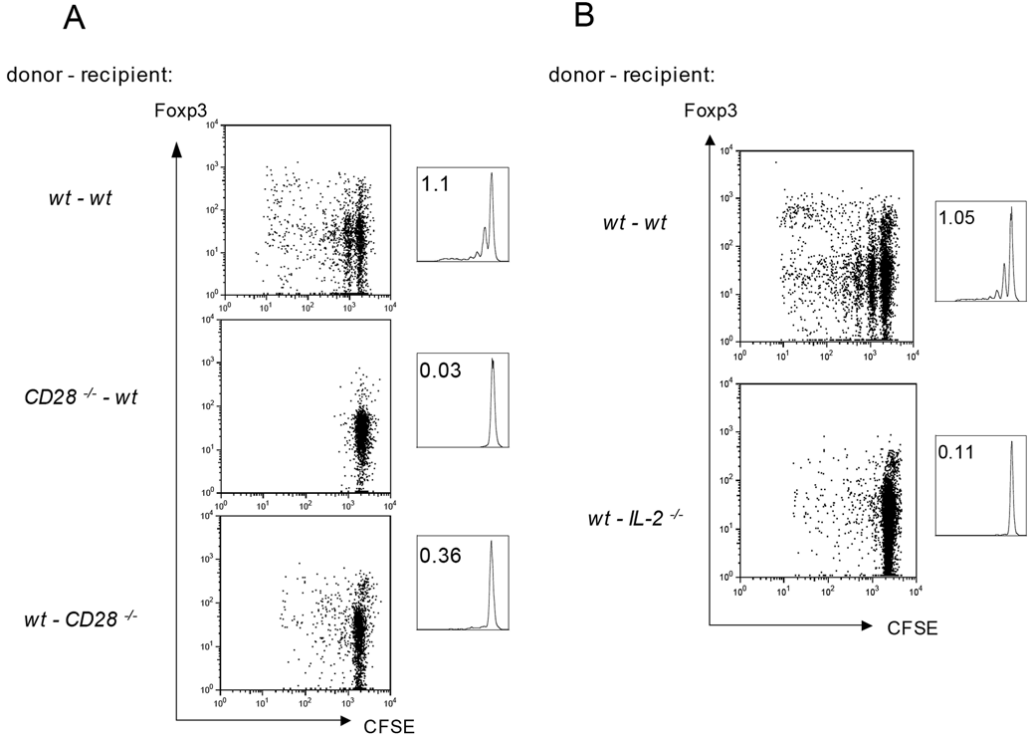
CD28SA mediated CD4 T-cell activation requires CD28 signals in *cis* and *trans*. 10^7^ CFSE-labeled purified CD4 T-cells were transferred on day −1 i.v. into wild type or knockout mice as indicated, and analyzed 3 d after CD28SA application. (A) Requirement for CD28 on both donor and host cells for proliferation of transferred cells. (B) Host-derived IL-2 is required for proliferation of donor cells.

In antigen-driven immune responses, conventional CD4 T-cells utilize IL-2 as an autocrine growth factor, but also provide this cytokine to Treg cells in a paracrine fashion, thereby increasing their number and suppressive activity [9], [10], [24]–[27]. Accordingly, we were interested to see whether CD28SA-induced proliferation of conventional and regulatory CD4 T-cells *in vivo* is a cell-autonomous effect, or whether alternatively, it relies on signals, e.g. IL-2, received from other activated T-cells. First, we confirmed the requirement of the responding CD4 T-cells to be stimulated themselves via CD28. CFSE-labeled CD4 T-cells from wt or CD28 knockout mice (which contain about 10 and 2% Treg cells, respectively) were transferred to CD28 wild type mice, which were then challenged with the CD28SA and analyzed for proliferation 3 days later. As seen in Fig. 3A, wt CD4 T-cells (both conventional and Treg), proliferated when stimulated by CD28SA within a wt host, whereas CD28 deficient donor cells failed to divide. In the converse experiment, i.e. transfer of wt CD4 T-cells into CD28 deficient hosts, proliferation of both subsets was also impaired, pointing at the importance of bystander-derived signals.

To investigate whether IL-2 is such an essential bystander-derived signal, wt CFSE-labeled CD4 T-cells were transferred to wt or IL-2 deficient hosts. Indeed, CD28SA induced hardly any proliferation of conventional or regulatory CD4 T-cells in IL-2 deficient recipients, demonstrating the requirement for paracrine IL-2 in the CD28SA-driven expansion of both subsets (Fig. 3B).

### Treg cells prevent systemic cytokine release in CD28SA treated mice

The dependence of CD28SA-induced CD4 T-cell proliferation on paracrine IL-2 indicated that there had to be significant IL-2 production to drive the CD28SA response. Accordingly, sera from CD28SA-stimulated mice were analyzed for the presence of circulating IL-2 at 2, 4 and 24 hours after injection. However, only minute levels of IL-2 (about 25 pg/ml, as compared to 4000 in the TGN1412 trial [20]), were observed in the circulation of CD28SA-treated mice (Table 1). We also investigated the levels of other circulating cytokines (ILs 4, 5, 6, 10, 12; IFNγ and TNF) which had contributed to the syndrome observed in the human volunteers of the TGN1412 study [20]. The highest systemic level observed was for IL-6 at 2 hrs (168 pg/ml; compared to 3500 in the TGN1412 study). In further experiments, samples were collected as early as 20 min. and up to three days after application of CD28SA, without any evidence for pathophysiologically revelant levels of circulating cytokines (data not shown).

**Table 1:** Circulating cytokine levels after CD28SA injection.

Cytokines	Control level	Days after CD28SA injection		
		2 h	4 h	24 h
IL-2	ND	26.43±8.50	12.84±5.17	ND
IL-4	0.49±0.44	1.96±0.90	1.87±0.95	1.59±1.50
IL-5	0.11±0.18	1.11±0.20	4.91±1.68	25.97±3.67
IL-6	1.71±1.62	168.16±54.67	125.09±72.06	7.21±3.61
IL-10	0.21±0.16	9.86±3.48	78.15±54.44	6.94±10.01
IL-12p70	0.13±0.23	ND	0.88±1.53	15.21±18.13
IFNγ	0.42±0.37	3.66±3.41	1.08±0.12	2.28±0.32
TNF	ND	27.97±24.73	56.07±71.20	ND

Groups of three mice received a single injection of 100 µg mAb D665. Mice were sacrificed at times indicated, and cytokines in sera were measured by cytokine bead array analysis. Values are given as pg/ml. ND = not detectable.

**Figure 4:**
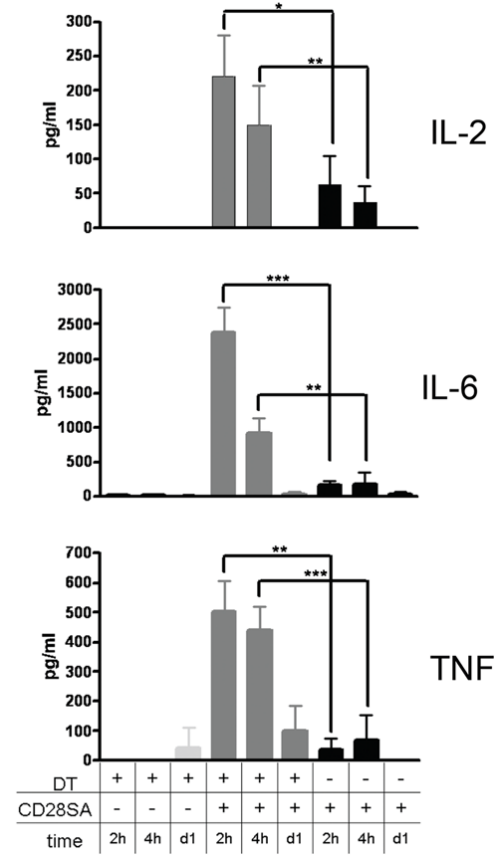
Treg depletion results in systemic cytokine release after CD28SA stimulation. Mice were pretreated with DT to remove Treg cells from DEREG mice as given in Methods, and stimulated with CD28SA for the times indicated. Cytokine levels in peripheral blood are shown.

One plausible explanation [6] for the absence of systemic cytokine release in CD28SA treated mice could be instantaneous consumption of IL-2 by Treg cells, which would contribute to their CD28SA-driven activation and result in the suppression of further cytokine production by the conventional CD4 T-cells. We tested this hypothesis by depletion of Treg cells from “DEREG” mice, which express the human diphteria toxin (DT) receptor as well as GFP under the control of the Foxp3 promoter, using DT [28]. We preferred this approach over CD25-directed mAb depletion because residual mAb would have influenced the amount of free IL-2 due to its blockade of IL-2R. As compared to the PBS treated control group where Treg cells comprised 12.7±0.92 percent of CD4 T-cells, DT-treated DEREG mice retained only 0.63±0.19 percent. As seen in Figure 4, serum samples obtained from these Treg depleted, CD28SA treated mice after 2 and 4 hours revealed significant levels of circulating IL-2 (200 pg/ml), IL-6 (2300 pg/ml) and TNF (500 pg/ml), which had disappeared by 24 hours. Of note, IFNγ and ILs 4, 10 and 12 remained low, while moderate amounts of IL-5 were detected (not shown). In contrast, no cytokine release was induced in PBS treated DEREG mice (Fig. 4) and in WT mice treated with DT or PBS (not shown). In conclusion, Treg depletion reveals the ability of CD28SA to trigger the release of pro-inflammatory cytokines also in mice, with the exception of IFNγ and IL-12.

### Treg activation is resistant to corticosteroid treatment

**Figure 5:**
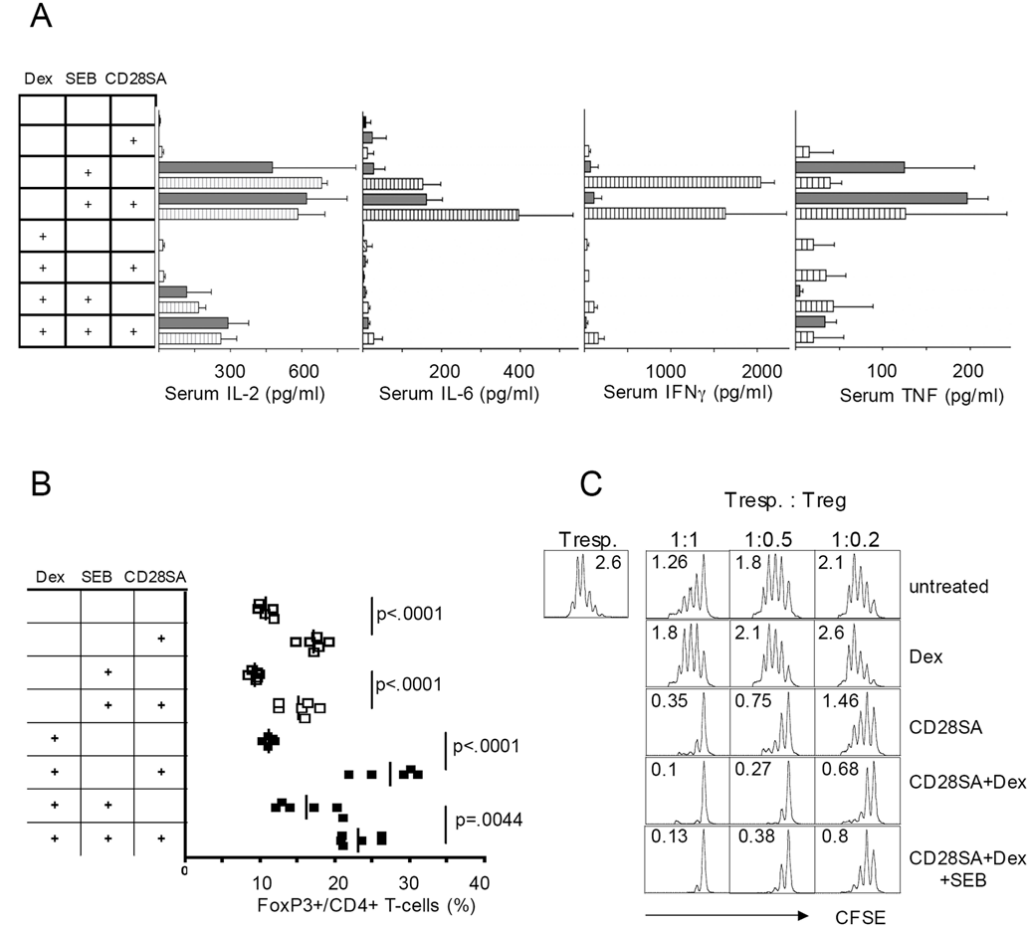
CD28SA mediated Treg activation is compatible with anti-phlogistic corticosteroid therapy. (A) Dexamethasone (Dex) effectively suppresses cytokine release induced by the superantigen SEB in the presence or absence of CD28SA stimulation. Sera of mice treated as indicated (see also Methods) were analyzed 1 hr (closed bars) or 4 hrs (stripped bars) after stimulation with SEB and/or CD28SA. (B) Increase in Treg cells is unaffected by Dex treatment. Analysis on day 3 after single stimulation with SEB and/or CD28SA, and daily treatment with 5 mg/kg Dex. (C) CD28SA-induced enhancement of Treg activity is resistant to Dex treatment. Treg cells isolated from groups shown in (B) were cocultered with CFSE-labeled indicator cells as described in Methods. Cell division was measured on d3, and average numbers are given as inserts.

The very low levels of circulating cytokines, including IL-2, in CD28SA stimulated mice indicate that a “cytokine storm” is not a prerequisite for CD28SA-driven Treg activation. Since in humans, however, application of the superagonist TGN1412 resulted in a rapid toxic cytokine release, we asked whether its pharmacological prevention would also interfere with the intended response, i.e. transient Treg expansion and activation. To test this in the mouse system, we induced systemic cytokine release with the bacterial superantigen SEB [29], in the presence or absence of 5 mg/kg/day of the corticosteroid dexamethasone (Dex), and studied the effects on concommitant stimulation with the CD28SA. To accommodate the rapid kinetics of TNF release in this system [29], measurements were taken at 1 and 4 hours. As shown in Figure 5A, high levels of IL-2, IL-6, IFNγ and TNF were observed in SEB-treated mice irrespective of simultaneous application of the CD28SA. No significant changes were detected for ILs 4, 5, and 12 (not shown). Pretreatment with Dex three hours before SEB injection reduced the IL-2 response by 3–5 fold, the IL-6 response by 10 fold, the IFNγ response 40 fold, and the TNF response by 5–10 fold, indicating successful suppression of cytokine release by corticosteroid prophylaxis.

Next, we asked whether CD28SA-driven Treg expansion and functional activation were also impaired by Dex treatment. In keeping with the well-established effects of corticosteroids on the peripheral immune system, we observed a clear reduction in total cellularity of spleen and lymph nodes (not shown). When analyzed on day 3 after stimulation, the frequency of nTreg cells among CD4 T-cells was enhanced, however, rather than reduced, by coadministration of Dex to CD28SA-treated mice (Fig. 5B). This effect was not observed in mice receiving Dex only, but was preserved in mice receiving SEB in addition to CD28SA and Dex.

We then titrated purified Treg cells from stimulated and naïve mice into a standard suppression assay employing conventional CD4 responder cells, irradiated APC and anti-CD3 as a T-cell mitogen (Fig. 5C). In all cases where the CD28SA had been applied *in vivo*, and irrespective of additional *in vivo* treatment with SEB or anti-phlogistic therapy with Dex, the Treg cells recovered were five to ten-fold superior in suppressive activity as compared to Treg cells from naïve mice. Thus, prophylactic treatment of mice with corticosteroids at doses which efficiently control a potential “cytokine storm” does not impair the Treg-promoting activity of CD28SA.

## Discussion

Our present results using a mouse-anti mouse CD28SA confirm the preferential expansion and activation of Treg over conventional CD4 T-cells initially reported in rats [17], [19], and suggest a mechanism for this effect: CD28SA-triggered activation of conventional CD4 T-cells provides a source of IL-2, which synergizes with the effect of CD28SA on the Treg cells themselves, thereby supporting their expansion and functional activation. This model [6] transfers the established IL-2 dependent regulatory circuit described for antigen-driven T-cell responses [9], [10], [24]–[27] to the polyclonal stimulus provided by CD28SA, with the major modification of a more rapid onset of counter-regulation which dampens the conventional CD4 T-cell response before it reaches the effector phase. Most likely, one of the decisive mechanisms in this regulatory circuit is the consumption of IL-2 by the CD28SA activated Treg cells [30], [31], leading to growth-factor withdrawal and apoptosis [32] of the effector cells, and to further functional activation of the Treg cells. Indeed, we found a dramatic upregulation of Treg effector function in CD28-stimulated mice which, together with their numeric expansion, well explains their anti-inflammatory effects (Fig. 2A). In agreement with previous studies performed in rats [19], [23], this hyperactivation of the regulatory T-cell compartment translates into protection from and interference with EAE (Fig. 2B). Of note, amelioration of inflammatory liver disease in a mouse model of African trypanosomiasis [33] has recently added another example to the list of rodent immunopathologies that are effectively treated by CD28SA-mediated Treg activation.

A key finding of the present study is the rapid release of substantial amounts of TNF, IL-6 and IL-2 into the circulation of CD28SA-treated mice which had previously been depleted of Treg cells, whereas no significant cytokine release is observed in CD28SA-stimulated control animals (Fig. 4, Table 1). This indicates that in Treg-sufficient rodents, Treg cells quickly get the upper hand and prevent a “cytokine storm”. In keeping with these results and our previously published model [6], a transient induction of cytokine mRNA, but no systemic cytokine release, was recently reported for CD28SA treated rats [34].

In the human volunteers of the TGN1412 study, the need for intensive medical intervention which, *inter alia*, included IL-2Rα-specific mAb, precluded further analysis of CD28SA effects on Treg cells. It is obvious from the unfortunate outcome of the trial, however, that in contrast to rodent models, Treg cells did not rapidly dominate the response of effector T-cells. The reasons for this difference in overall reactivity of the human and rodent immune systems remain elusive in spite of a number of suggestions (reviewed in [35]). This holds equally true for the difference in reactivity between the cynomologus monkeys used in preclinical testing of TGN1412 itself, which had well tolerated the antibody [36], and the human volunteers.

While it is conceivable that more than one mechanism is involved in the marked species differences of the response to CD28SA, the question is worth asking whether pharmacologic suppression of systemic cytokine release is compatible with the desired effect of the mAb, i.e. transient polyclonal Treg activation. There is ample experience with this type of intervention [37] in mAb therapy because cytokine release syndromes such as observed with TGN1412 are not unique to this type of mAb but rather have been observed with other agonistic mAb such as anti-CD3 [38], [39], but also with blocking or depleting mAb to cell surface receptors not thought to mediate activating signals [40]. Therefore, prophylactic and interventional protocols have been developed to contain such “cytokine storms” which employ corticosteroids as their key component. These effectively curb the release of cytokines with toxic systemic activity such as TNF, IFNγ, IL-6 and IL-2, all of which very rapidly reached dramatic levels in the TGN1412 volunteers [20].

Since mice do not respond to CD28SA with significant systemic cytokine release (Table 1) as a result of rapid Treg activation, we tested the effects of corticosteroids in mice additionally stimulated with a bacterial superantigen [29]. Our results (Fig. 5) show that superantigen-mediated activation of effector-cells with the potential to release systemic pro-inflammatory cytokines, and the simultaneous suppression of this cytokine release by corticosteroid prophylaxis do not interfere with CD28SA-mediated Treg activation. Interestingly, while the circulating levels of IFNγ, TNF and IL-6 as crucial mediators of immunopathogenesis were almost completely suppressed by Dex, that of IL-2 was only 3–5 fold reduced suggesting that the effects of corticosteroids on IL-2 production stimulated by CD28 would have left sufficient “fuel” for the activation of Treg cells. On the side of the Treg cells themselves, it has indeed been shown in mice that their activation by systemic infusion of IL-2 is resistant to corticosteroid therapy [5].

In summary, our findings indicate that the cytokine storm observed in the TGN1412 trial was not detected in preclinal rodent models due to the rapid control of CD28SA-induced cytokine release by Treg cells. This Treg cell response is itself triggered by CD28SA, in keeping with the important role of CD28 signalling in Treg homeostasis and activation, but also depends on CD28SA stimulation of conventional T-cells, which provide paracrine IL-2. The dependence of Treg activation on paracrine cytokine delivery does, however, not preclude the use of corticosteroids to suppress of pro-inflammatory cytokine release, which provides an important safety shield in mAb therapies.

On a more general note, the marked ability of Treg cells to prevent systemic cytokine release in mice should be taken into account in current and future preclinical rodent studies of immunomodulatory biopharmaceuticals. In practice, toxicity testing in Treg depleted mice may be helpful in predicting such a possible inflammatory response.

## Materials and Methods

### Ethics statement

All animal experimentation was performed under permit by the Government of Lower Franconia in accordance with the state regulations guidelines for animal welfare.

### Mice

C57BL/6 and BALB/c mice purchased from Harlan Winkelmann (Borchen, Germany). Congenic Thy1.1 mice and CD28^−/−^ mice were from Jackson Laboratories. IL-2-deficient mice [41] on a C57BL/6 background were derived at the Institute, and DEREG mice [28] were generously provided by Tim Sparwasser. Mice between 6–10 weeks of age were used and maintained in the institute's barrier-facility.

### MAb treatment

MAb D665 [22] was bioreactor-produced by Exbio, Praha, Tchec Republic, Invivo Biotech, Henningsdorf, Germany, or Serotec, Oxford, UK. PV-1 was used as negative control. All preparations were in a low-endotoxin format and injected i.p. Indistinguishable results were obtained with i.v. injection. Unless stated, 100 µg/mouse were used.

### EAE induction and treatment with CD28SA *in vivo*

Active EAE was induced in C57BL/6 mice by immunization with 50 µg MOG_35–55_ (Institute of Medical Immunology, Charité, Berlin, Germany) in PBS emulsified in an equal volume of CFA containing Mycobacterium tuberculosis H37RA (Difco, Detroit MI, USA) at a final concentration of 1 mg/ml. 200 ng pertussis toxin (List Biochemicals, Campbell, CA, USA) were given i.p. on day 0 and 2. Mice were scored daily according to a 10 point scale[42]. CD28SA was given as a single i.p. injection of 50 µg on the day of immunization (preventive setting) or on day 9 (therapeutic setting). In some experiments, Treg were depleted by 6 i.p. injections of 100 µg anti-CD25 (clone PC61) every third day followed by EAE immunization using 25 µg MOG_35–55_.

### Induction of systemic cytokine release and corticosteroid treatment

BALB/c mice received a single intravenous injection of 50 µg of bacterial superantigen - SEB (Staphylococcal Enterotoxin B). Additionally they were stimulated with 200 µg of CD28SA. This was performed in the presence or absence of 5 mg/kg dexamethasone (Dex) pre-treatment (3 hours before SEB/CD28SA), with subsequent daily injections of Dex.

### Antibodies used for flow cytometric analyses and for purification of T-cells

CD4 (RM4-5), CD25 (PC61 or 7D4), CD8 (53–6.7), CD3 (17A2), B220 (RA3-6B2), Thy1.1 (OX-7). CD11b (M1/70), CD49b (Dx5), B220 (RA3-6B2), TER-119 (TER-119): BD Pharmingen, Foxp3-(FJK-16s): eBioscience.

### Cytokine detection

For detection of serum cytokines ELISA sets (OptEIA™, BD Pharmingen) and Cytometric Bead Array (CBA, BD Pharmingen) were used according to the manufacturer's instructions.

### Flow cytometry

Single cell suspensions were incubated with anti-CD16/CD32 mAb (2.4G2; Fc block, BD Pharmingen) followed by staining of extracellular markers. Foxp3 analysis was performed according to the manufacturer's instructions (eBioscience). Acquisition performed on a FACSCalibur™ and data were analyzed using FlowJo software (Beckton Dickinson).

### Purification of T-cells

Single-cell suspensions were stained with a cocktail of biotin-labeled antibodies, followed by incubation with Streptavidin MicroBeads. CD4^+^ T-cells were prepared by negative selection using the MACS separation system (Miltenyi Biotech). CD25^+^ and CD25^−^ CD4 T-cells were separated using PE labeled anti-CD25 mAb and anti-PE- MicroBeads. Purity of cells was 90–95%.

### CFSE-labeling and Cell Transfer

Purified CD4 T-cells were labeled with 10 µM CFSE at RT for 5 min. 10^7^ cells in PBS were transferred i.v. one day before CD28SA stimulation.

### Treg cell depletion

DEREG mice were treated daily with intraperitoneal injection of diphteria toxin (DT; 1 µg) for five days before administration of CD28SA. Control groups received PBS instead of DT or CD28SA. Efficiency of depletion was monitored by flow cytometric analysis of GFP positive cells.

### Suppression Assay

Isolated CD4^+^CD25^−^ responder T-cells (5×10^4^) were CFSE labeled and cultured for 3 days in U-bottomed 96-well plates with a various dilutions of purified CD4^+^CD25^+^ Treg cells in the presence of soluble anti-CD3 (1 µg/ml) and irradiated splenic APC (20Gy; 2×10^5^). Proliferation was assayed by CFSE dilution.

### Statistical analysis

Statistical significance for normal distributed samples was analyzed by unpaired *t*-test. Data with unequal variances were tested with Mann-Whitney rank sum test using GraphPad Prism Software. Values of p<0.05 were considered significantly significant.
